# High-risk human papillomavirus diversity among indigenous women of western Botswana with normal cervical cytology and dysplasia

**DOI:** 10.1186/s12879-024-10058-z

**Published:** 2024-10-15

**Authors:** Patricia S. Rantshabeng, Billy M. Tsima, Andrew K. Ndlovu, Keneilwe Motlhatlhedi, Kirthana Sharma, Carol B. Masole, Natasha O. Moraka, Kesego Motsumi, Angela K. T. Maoto-Mokote, Alemayehu B. Eshetu, Leabaneng Tawe, Tendani Gaolathe, Sikhulile Moyo, Lynnette T. Kyokunda

**Affiliations:** 1 Department of Pathology, Faculty of Medicine, University of Botswana, Gaborone, Botswana; 2 School of Allied Health Profession, Faculty of Health Sciences, University of Botswana, Gaborone, Botswana; 3 Department of Family Medicine, Faculty of Medicine, University of Botswana, Gaborone, Botswana; 4 Botswana- Harvard Health Partnership, Gaborone, Botswana; 5 Rutgers Global Health Institute, Paterson, St New Brunswick, 112 USA; 6 Department of Internal Medicine, Faculty of Medicine, University of Botswana, Gaborone, Botswana; 7 Botswana-UPenn Partnership, Gaborone, Botswana; 8 Department of Immunology and Infectious Diseases, Harvard T.H. Chan School of Public Health, Boston, MA USA; 9 School of Health Systems and Public Health, Faculty of Health Sciences, University of Pretoria, Pretoria, South Africa; 10 Division of Medical Virology, Faculty of Medicine and Health Sciences, Stellenbosch University, Cape Town, South Africa

**Keywords:** High-risk HPV genotypes, Cervical lesions, Indigenous, Marginalized, Botswana

## Abstract

**Background:**

Cervical cancer remains a public health problem despite heavy global investment in health systems especially in low-and-middle-income countries (LMIC). Prophylactic vaccines against the most commonly detected human papillomavirus (HPV) types in cervical cancers are available and decisions on the selection of vaccine design depends on the prevalence of high-risk (hr) HPV genotypes for a particular region. In 2015, Botswana adopted the use of a quadrivalent HPV vaccine as a primary prevention strategy. Secondary prevention includes cervical smear screening whose uptake remains notably low among indigenous and marginalized communities despite efforts to improve access.

**Aim:**

To determine the prevalence of hrHPV genotypes and cervical lesions’ burden in women from the indigenous and marginalized communities of Botswana.

**Methods:**

This prospective survey enrolled 171 non-HPV vaccinated women aged 21 years and older. Face-to-face interviews, Pap smear screening, hr-HPV and Human Immuno-deficiency virus (HIV) testing were carried out. Conventional Papanicolau smears were analyzed and cervical brushes were preserved for hrHPV testing using the Ampfire Multiplex HR-HPV protocol which detects the following genotypes: HPV 16, 18, 31, 35, 39, 45, 51, 52, 53, 56, 58, 59 and 68.

**Results:**

In this study, 168/171 (98.6%) of the women consented to HIV testing; 53/171 (31%) were living with HIV and self-reported enrolment on antiretroviral therapy. Among the women examined, 23/171 (13.5%) had cervical dysplasia with most presenting with Atypical Squamous Cells of Undetermined Significance 8/23 (35%), Low-Grade Squamous Intraepithelial Lesions 8/23 (35%), Atypical Squamous Cells-High Grade 4/23 (17%), Atypical Endocervical Cells 2/23 (9%) and Atypical Endocervical Cell favoring neoplasia 1/23(4%). However, no High-Grade Squamous Intraepithelial Lesions (HSIL) or squamous cell carcinoma (SCC) were detected. Overall hrHPV prevalence in this study was at 56/171 (32.7%). The most commonly detected hrHPV genotypes in women with cervical dysplasia were HPV39 (6.25%), HPV51 (14.5%), HPV52 (12.5%) and HPV56 (4%). Notably, HPV 16 and 18 were not found in women with cervical dysplasia.

**Conclusions:**

Our study provides valuable insights into the prevalence and distribution of hrHPV genotypes in indigenous and marginalized communities in Botswana, and the need for further investigation of their potential role in cervical carcinogenesis in this population. These results may also serve as baseline data to facilitate future evaluation of the HPV vaccine needs.

## Background

Cervical cancer is the leading cause of cancer morbidity and mortality in women from low- and middle-income countries (LMICs) [1, 2]. Cervical cancer is etiologically linked to persistent genital infection with several oncogenic high-risk (hr) HPV genotypes which include, HPV 16, 18, 31, 33, 35, 39, 45, 51, 52, 56, 58, 59, 66, and 68 [3]. According to the working group of the World Health Organization (WHO) International Agency for Research on Cancer (IARC), the most commonly detected HPV genotypes (16, 18, 31,51, 52, 53, 58 and 68) are Group 1 carcinogens [4]. Prophylactic vaccines targeting some common hrHPV genotypes are available and while their potency has been demonstrated, vaccination rates remain low in many LMICs [5, 6]. It is well established that cervical cancer is preceded by a premalignant phase that lasts approximately 10 to 15 years, hence it is preventable and curable with adequate, comprehensive, regular organized community screening and prompt treatment of detected cervical lesions [2]. Therefore, measures to curb cervical cancer burden should continue to enforce improved effective prevention of hrHPV via vaccination and timely detection of associated premalignant cervical lesions [7]. Worth noting is that the prevalence of non-vaccine targeted hrHPV genotypes warrant the need for continued screening efforts for pre-neoplastic cervical lesions surveillance [8].

Botswana, like many other countries in sub-Saharan Africa, has a high HIV prevalence [9]. In addition, cervical cancer is the leading malignancy in both mortality and morbidity among women in Botswana [1, 10]. Botswana ranks fourth highest in adult HIV prevalent countries globally after South Africa, Eswatini and Lesotho [9]. HIV infection has been reported to contribute to HPV acquisition and more rapid cancer progression of cervical dysplastic lesions contributing to poorer patient outcomes [11, 12]. Studies have also reported that despite the introduction of highly active antiretroviral therapy (HAART), HPV-associated cervical cancer cases continue to rise [13, 14]. A study by Dryden-Peterson et al., [15] reported an annual increase in HPV-associated malignancies in people living with HIV in Botswana, despite a successful antiretroviral therapy program. To address the need for continued cervical cancer lesion development surveillance, HPV vaccination, effective screening and prompt treatment of cervical lesions are urgently needed for women living with HIV (WLWH), [16].

The HPV vaccination program in Botswana began in 2015, with the quadrivalent HPV vaccine (HPV 6/11/16/18) targeting all schoolgirls aged 9 to 13 years [17] and is reaching almost 100% coverage of the targeted population [18]. The only hrHPV genotypes targeted through this vaccination drive are HPV 16 and 18. Indigenous and marginalized communities of Botswana are located in Kgalagadi North and Ghanzi Districts and are traditionally a hunting and nomadic community, values that they still cherish today. However, in the 1990s, the government of Botswana embarked on a settlement program, building designated communities to enable better service provision: construction of shelter for elderly persons, provision of food rations, clothing, water and health care services and education. Although these services are provided, indigenous communities still prefer their century-old practices regarding health, food and culture [19, 20]. Indigenous communities tend to prefer their traditional practices for treatment and prevention of disease [21]. The strong cultural beliefs they hold dear make them less inclined to uptake modern healthcare services which are further compounded by the long distances needed to travel to secondary healthcare facilities for delivery and other services not available at the primary healthcare points with hardly any public transport facilities available [22]. The government of Botswana has achieved remarkable success in exceeding the UNAIDS 95–95-95 target: 95% of all individuals living with HIV are aware of their status, 95% of those diagnosed are receiving sustained antiretroviral therapy (ART), and 95% of those on ART have achieved virologic suppression. The current coverage rates for Botswana stand at 95.1%, 98%, and 97.9%, respectively [23–25]. Cervical cancer screening and management using the Pap smear and its alternative, “see and treat” program for cervical cancer lesions using visual inspection with acetic acid, is also well established [25–29].

While cervical cancer screening tests such as the Pap smear have been successful in reducing cervical cancer incidence and HPV vaccination expected to further reduce HPV infection and related cancers [30], the true extent of cervical disease burden in indigenous communities especially in LMIC [30], remains largely unknown and underestimated due to lack of data emanating from low screening rates [31]. Therefore, in order to achieve the World Health Organization (WHO) goals for elimination of cervical cancer in all women, these health inequities must be addressed, starting with generation of data on the impact of these initiatives in reducing the burden of cervical cancer, particularly in the indigenous and marginalized communities. There is paucity of cervical cancer screening data from indigenous populations in Botswana, with a few studies having been conducted among women from urban areas, who have access to more organized healthcare.

To address this, our study was designed to determine the prevalence of hrHPV genotypes and the burden of cervical pre-malignant and malignant disease in women from isolated indigenous populations in Botswana.

## Methods

### Study design and population

**Fig. 1:**
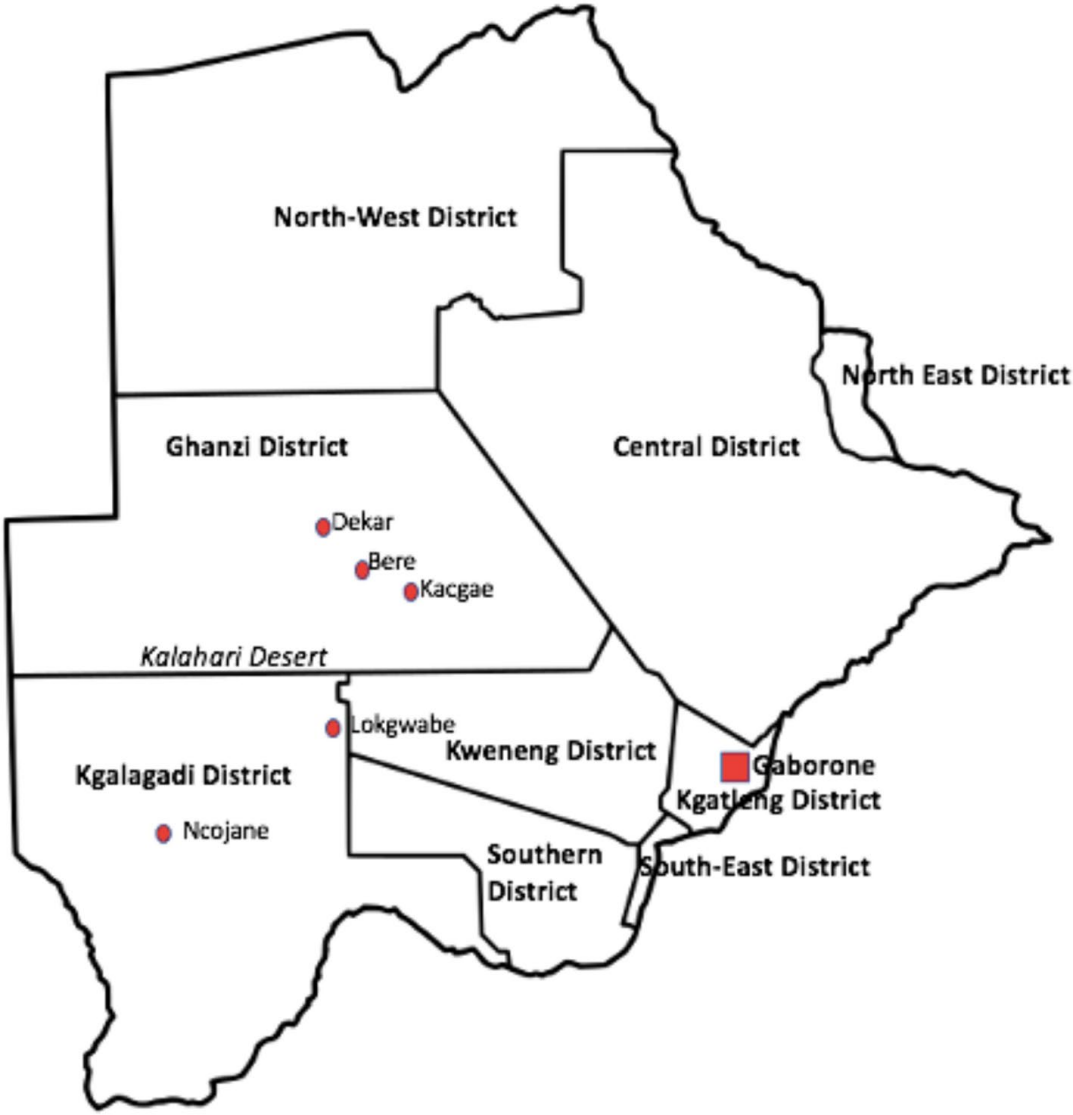
The map of Botswana showing the 5 study sites located in the Ghanzi and Kgalagadi districts: Kacgae, Bere, Lokgwabe, Dekar highlighted in red dots. Gaborone, the capital city where the National Health Laboratory (NHL) and University of Botswana are located, is depicted by a red square*. (Map credit: Patricia S. Rantshabeng)*

A prospective community survey was undertaken from June 6th to October 18th, 2022, enrolling a total of 171 consenting women aged 21 to 85 years. This study excluded women with a history of hysterectomy and pregnant women. Participants were recruited from five communities (Fig. 1): 3 settlements and 2 small villages of indigenous and marginalized communities. According to the Botswana 2022 Population and Housing census, the settlements and villages chosen for the study had the following populations: Kacgae -776 (380 males & 366 females), Bere – 874 (438 males & 436 females), Dekar -2814 (1382 males & 1432 females), Lokgwabe -1792 (879 males & 913 females), and Ncojane had 2242 (1045 males & 1197 females) people, [32].

#### Study sites

Recent estimates indicate that there are approximately 68 000 people in Botswana who identify as indigenous (marginalized). Many of them have been relocated to settlements in the Ghanzi and Kgalagadi districts. These settlements are remote and found in the Kgalagadi desert, joined together by the Trans Kalahari highway through tarmac and gravel roads which necessitates the use of off-road vehicles for easier access due to limited public transportation. Pap smears collected from women in this region are transported to the NHL in Gaborone, a referral and public health laboratory offering specialist laboratory services including Anatomical Pathology. The NHL also receives samples from the private health sector, with an annual average of 15,000 Pap smears received and resulted. The shortest distance from the study sites to the NHL was from Kacgae settlement (540 km), and the furthest distance was from Ncojane clinic (991 km). The average shortest distance from the cervical cancer screening and treatment facilities was 42 km from Dekar settlement to the nearest See & Treat clinic in Ghanzi. Bere and Kacgae settlements also referred patients to the Ghanzi-based clinic which is 154 km and 172 km respectively for each. Due to limited public transportation in Bere and Kacgae, most patients use the health post ambulance to access the Ghanzi-based care facility. Patients from Lokgwabe access the Kang-based See & Treat clinic which is 120 km away from the village. The furthest study site was Ncojane, which is 385 km from the Ghanzi-based care facilities and requires patients to have lodging arrangements when traveling for treatment.

#### Data and sample collection

Each community health post/clinic was used as a study site for data and sample collection. Face-to-face interviews using a structured questionnaire developed specifically for this study (see supplementary files), were used to collect demographic data such as age, sexual history, cervical screening history, tobacco and alcohol use history, parity and contraceptive use. Conventional Pap smear collection and rapid HIV testing was conducted by trained research nurses and a medical doctor for the study. The hrHPV samples were collected from cervical smear collection brushes, post smearing and preserved in 10 ml of phosphate buffered saline (PBS). Cervical brushes were preserved at room temperature. All samples collected were transported and analyzed in Gaborone, Botswana.

### Rapid HIV testing

Rapid HIV testing was performed using the Maxline HIV 1 & 2 Tri-Line Rapid Test manufactured by Avecon Healthcare Pvt Ltd (Haryana, India-133104). Following aseptic procedures and sanitisation with 70% alcohol swab, a needle prick was made on a participants’ finger to obtain a blood specimen for the HIV test. A blood sample of about 0.5 ml was obtained and placed on the test strip followed by adding a buffer (as per the manufacturer’s instruction), and incubated for 15 min at room temperature. The test was interpreted as negative if only one line appeared on the control test strip, and positive if two lines appeared on both the control and test strips. Pre- and post-testing counseling was conducted by a trained research nurse before results were dispatched on site, to the participants.

### Pap smear analysis

The Pap smear slides were stained in-house at the University of Botswana School of Allied Health Sciences laboratory using routine Papanicolaou stain according to the approved quality control and assurance procedures. Preliminary Pap smear screening was done by a Cytotechnologist and reported following the 2014 Bethesda system for reporting cervical cytology. As part of the quality assurance, the results were reviewed and verified by two Anatomical Pathologists to ensure agreement. All specimens collected were adequate for evaluation. Results for Pap smear analysis and hrHPV testing were returned in a timely manner on the next visit and hand-delivered to participants by the study team together with their health post/clinic nurse. Participants with abnormal lesions were linked to the nearest cervical cancer treatment facility for treatment and further management through their healthcare facility (study site).

### High-risk HPV sample preparation, detection and genotyping

Samples were centrifuged at 3500 rpm for 5 min to dislodge the cells from the collection brushes and aliquoted into 1.5 ml eppendorf tubes prior to analysis. Each sample was mixed briefly by vortexing and was concentrated by a centrifugation step, for 30 min at a maximum 3500 rpm. After centrifugation, the supernatant was removed completely and 30 μl of the lysis buffer was added to the tube. The pellet and lysis buffer were then thoroughly mixed using a vortex to resuspend the cell pellet. The resuspended pellet was transferred to a PCR tube, which was incubated at 95 °C for 10 min and immediately used for hrHPV detection after the lysis step. High-risk HPV detection was achieved by using the Atila AmpFire® Multiplex HPV Assay (AmpFire) manufactured by AtilaBioSystems (Mountain View, CA, USA). This is an in vitro nucleic acid isothermal amplification with real-time fluorescence detection assay for the qualitative genotyping of hrHPV genotypes 16, 18, 31, 33, 35, 39, 45, 51, 52, 53, 56, 58, 59, 66 and 68 from different sample types. The assay uses 4 primer mixes targeting the 15 hrHPV genotypes and has 4 channels (FAM/HEX/ROX/CY5) and specific gene targets which are fluorescently labelled and generate target amplification signals in real-time over 60 min. This assay also uses an internal control targeting the human- β-globin gene for each sample. The lack of amplification curve in the HEX was regarded as invalid and the test has to be repeated. All samples had a detectable internal control and were deemed adequate for analysis.

### Statistical analysis

Statistical analysis was performed using R-statistical software version 4.3.1. A sample size of 171 women was calculated as sufficient to determine the burden of disease and hrHPV infection. Owing to their cultural diversity and isolated living arrangements using a power of 0.8 alpha 0.05, with a proportionate sampling of 2 small villages and 3 settlements of differing population sizes provided a population representative sample. Type-specific hrHPV prevalence was calculated with a 95% confidence interval (CI) among women enrolled in the study. Fisher’s Exact tests and Pearson’s Chi-square were used to determine the association between categorical variables and statistical significance was determined by a p-value of ≤ 0.05.

## Results

### Characteristics of the study population and hr-HPV detected in the study

A total of 171 female participants were enrolled in the study, with ages ranging from 21 to 85 years (mean age 41.5). Among the cohort, 74% (127/171) of the women were ≤ 50 years while 26% (44/171) were > 50 years of age. About 4% (7/171) of the women reported having no children, 16% (28/171) had one child and 80% (136/171) reported having had more than one child in their lifetime. Fifteen percent 15% (25/171) of the women reported having one lifetime sexual partner and 85% (146/171) reported having multiple partners, with a mean age of sexual debut at 18.5 years. The majority of the women (61%) were using hormonal contraceptives at the time of data collection. In addition, 17% of the women were current smokers, and 8.8% had a history of smoking (ex-smokers). High risk HPV was detected in 56/171 (32.7%) participants. Fifty-three out of one hundred and seventy-one (31%) women were living with HIV and self-reported enrolment in the national ART program.

The socio-demographic characteristics and contraception use history of the study participants stratified by hrHPV DNA results are presented in Table 1.

**Table 1:** Characteristics of the participants and hrHPV detection in the study

hrHPV DNA detection				
	**Overall (***n*** = 171)**	**Detected (***n*** = 56)**	**Not detected (***n ***= 115)**	*P***-value**
**Age**				
Mean (SD)	41.5 (12.4)	41.9 (11.6)	41.3 (12.8)	0.75
**Age at 1st coitus**				
Mean (SD)	18.5 (3.16)	18.5 (3.03)	18.5 (3.24)	0.98
**HIV status**				
Positive	53 (31%)	18 (32.1%)	35 (30.4%)	0.06
Negative Unknown	115 (67.25%) 3(1.75%)	35 (62.5%) 3 (5.4%)	80 (69.6%) 0	
**Lifetime # of sexual partners**				
1 partner	25 (14.6%)	9 (16.1%)	16 (13.9%)	0.82
More than 1 partner	146 (85.4%)	47 (83.9%)	99 (86.1%)	
**Smoking history**				
Current smoker	29 (17.0%)	18 (32.1%)	11 (9.6%)	0.001
Non-smoker	127 (74.3%)	34 (60.7%)	93 (80.9%)	
Ex-smoker	15 (8.8%)	4 (7.1%)	11 (9.6%)	
**Alcohol use**				
Yes	56 (32.7%)	23 (41.1%)	33 (28.7%)	0.08
No	74 (43.3%)	25 (44.6%)	49 (42.6%)	
Stopped	41 (24.0%)	8 (14.3%)	33 (28.7%)	
**Family history of cancer**				
Yes	51 (29.8%)	14 (25.0%)	37 (32.2%)	0.38
None	120 (70.2%)	42 (75.0%)	78 (67.8%)	
**Parity**				
0	7 (4.1%)	3 (5.4%)	4 (3.5%)	
1	28 (16.4%)	9 (16.1%)	19 (16.5%)	0.85
> 1	136 (79.5%)	44 (78.6%)	92 (80.0%)	
**Previous contraceptive**				
Condom only	24 (14.0%)	5 (8.9%)	19 (16.5%)	
Condom, injectables	2 (1.2%)	1 (1.8%)	1 (0.9%)	
Condom, pill	2 (1.2%)	1 (1.8%)	1 (0.9%)	
Condom, pill, injectables	3 (1.8%)	2 (3.6%)	1 (0.9%)	
Injectables only	56 (32.7%)	18 (32.1%)	38 (33.0%)	0.67
Loop, injectables	3 (1.8%)	2 (3.6%)	38 (33.0%)	
Pill only	27 (15.8%)	11 (19.6%)	16 (13.9%)	
Pill, injectables	21 (12.3%)	8 (14.3%)	13 (11.3%)	
Pill, injectables, loop	1 (0.6%)	0	1 (0.9%)	
Pill, loop	2 (1.2%)	1 (1.8%)	1 (0.9%)	
None	30 (17.5%)	9 (16.1%)	21 (18.3%)	

### Cervical cytology analysis and hrHPV results

A total of 23/171 (13.5%) women had abnormal cervical lesions as shown in Table 2. Among the entire cohort, 56/171 (32.7%) tested positive for hrHPV. Of the women with cervical premalignancies, 8/23 (35%) had hrHPV detected. Within this group, 4/23 (17.4%) had Atypical Squamous Cells-High Grade (ASC-H) with hrHPV detected in 1/4 (25%) (Table 2). In addition, 8/23 (35%) women had Atypical Squamous Cells of Undetermined Significance (ASCUS) with hrHPV detected in 4/8 (50%) of the study subjects. Atypical endocervical cells (AEC) were diagnosed in 2/23 (9%) of the women, and hrHPV was not detected in any of these participants. Only 1/23 (4%) of the women had Atypical Endocervical Cells favoring neoplasia (AEC-FN), however she tested negative for hrHPV. Furthermore, 8/23 (35%) of the women had Low-Grade Squamous Intraepithelial Lesions (LSIL) and 3/8 (37.5%) of them had hrHPV while 5/8 (62.5%) of them tested negative for hrHPV.

**Table 2:** Proportion of hrHPV DNA detected in final cytology

Cytology diagnosis	**hrHPV DNA**		
	**Positive**	**Negative**	**Total**
ASC-H	1 (25%)	3 (75%)	4 (100%)
ASCUS	4 (50%)	4 (50%)	8 (100%)
AEC	0	2 (100%)	2 (100%)
AEC-FN	0	1 (100%)	1 (100%)
LSIL	3 (37.5%)	5 (62.5%)	8 (100%)
NILM	48 (32.4%)	100 (67.6%)	148 (100%)
Total	56 (32.7%)	115 (67.3%)	171 (100%)

Fisher’s Exact test: *P*-value = 0.861

#### Distribution of hrHPV genotypes in cervical cytology

Of the 56/171(32.7%) women who tested positive for hrHPV, only 8/56 (14.3%) had cervical pre-malignant lesions. One with ASC-H had HPV58. Of the four women with ASCUS, two tested positive for HPV56, one for HPV35 and the other for HPV51. Three women had Low-grade squamous intraepithelial lesion (LSIL). All three women had co-infection of two hrHPV genotypes: one had HPV18 & HPV39, another had HPV31 & HPV52, while the other had HPV52 & HPV56 respectively. Additionally, 48 women did not have any cytological abnormalities and among those with single infections; six (12.5%) had HPV52, four (8.3%) had HPV31, four (8.3%) had HPV35, three (6.3%) had HPV16, three (6.3%) had HPV51, three (6.3%) had HPV58, three (6.3%) had HPV68, two (4.2%) had HPV18, two (4.2%) had HPV 33, two (4.2%) had HPV39, two (4.2%) had HPV45, two (4.2%) had HPV53, two (4.2%) had HPV66 and one (2.1%) had HPV59. Among those who had two or multiple hrHPV genotypes detected, two women (4.2%) had HPV 16 & 51, one (2.1%) had HPV 33 & 39, one (2.1%) had HPV52 & 53, one (2.1%) had HPV 53 & 68, one (2.1%) had HPV31 & 58, one (2.1%) had HPV18, 35 & 66, one (2.1%) had HPV 31, 35, 58 & 68 and one woman had HPV 16, 31, 51, 59, 45 & 53 co-infection (Table 3). A Pearson’s Chi-square: *P*-value = 0.61, Fisher’s Exact test: *P*-value = 0.352 showed that there was an insignificant relationship between hrHPV genotype and cytology results as shown in Table 3.

**Table 3:** The distribution of hrHPV genotypes according to cytology results

		Final cytology diagnosis				
		ASC-** H**	ASCUS	LSIL	NILM	TOTAL
hrHPV TYPE	16	0	0	0	3 (100%)	3 (100%)
	16, 31, 45, 51, 59, 53	0	0	0	1 (100%)	1 (100%)
	16, 51	0	0	0	2 (100%)	2 (100%)
	18	0	0	0	2 (100%)	2 (100%)
	18, 35, 66	0	0	0	1 (100%)	1 (100%)
	18, 39	0	0	1 (100%)	0	1 (100%)
	31	0	0	0	4 (100%)	4 (100%)
	31, 35, 58, 68	0	0	0	1 (100%)	1 (100%)
	31, 52	0	0	1 (100%)	0	1 (100%)
	31, 58	0	0	0	1 (100%)	1 (100%)
	33	0	0	0	2 (100%)	2 (100%)
	33, 39	0	0	0	1 (100%)	1 (100%)
	35	0	1 (20%)	0	4 (80%)	5 (100%)
	39	0	0	0	2 (100%)	2 (100%)
	45	0	0	0	2 (100%)	2 (100%)
	51	0	1 (25%)	0	3 (75%)	4 (100%)
	52	0	0	0	6 (100%)	6 (100%)
	52, 53	0	0	0	1 (100%)	1 (100%)
	52, 56	0	0	1 (100%)	0	1 (100%)
	53	0	0	0	2 (100%)	2 (100%)
	53, 68	0	0	0	1 (100%)	1 (100%)
	56	0	2 (100%)	0	0	2 (100%)
	59	0	0	0	1 (100%)	1 (100%)
	58	1 (25%)	0	0	3 (75%)	4 (100%)
	66	0	0	0	2 (100%)	2 (100%)
	68	0	0	0	3 (100%)	3 (100%)
	TOTAL	1 (1.8%)	4 (7.1%)	3 (5.4%)	48 (87.1%)	56 (100%)

Pearson’s Chi-square: *P*–value = 0.61, Fisher’s Exact test: *P*-value = 0.352

Table 4 presents the age distribution of study participants by hrHPV status and final cytology diagnosis. Among those detected with hrHPV (n = 56); there was 1 (0.58%) case of ASCUS and 9 (5.26%) cases of NILM in the 20–30 age group. For those with no hrHPV (n = 115), the 20–30 age group had 1 (0.58%) case of AEC, 2 (1.17%) cases of LSIL, and 19 (11.11%) cases of NILM. In the 31–40 age group there were 2 (1.17%) cases of LSIL and 9 (5.26%) cases of NILM among those with detectable hrHPV. For those with no detectable hrHPV, there was 1 (0.58%) case of ASC-H, 2 (1.17%) cases of ASCUS, 1 (0.58%) case of AEC-FN, 1 (0.58%) case of LSIL, and 31 (18.13%) cases of NILM. In the 41–50 age group, those with detectable hrHPV had 3 (1.75%) cases of ASCUS, 1 (0.58%) case of LSIL, and 16 (9.36%) cases of NILM. Those with no detectable hrHPV had 2 (1.17%) cases of ASC-H, 1 (0.58%) case of ASCUS, 1 (0.58%) cases of AEC, 2 (1.17%) cases of LSIL, and 22 (12.87%) cases of NILM. For participants aged 50 and above, those with detectable hrHPV had 1 (0.58%) case of ASC-H and 14 (8.19%) cases of NILM. In those without hrHPV, there was 1 (0.58%) case of ASCUS and 28 (16.37%) cases of NILM. Fisher’s exact test was used to assess the association between age groups, hrHPV status and cytology diagnosis.

**Table 4:** Age distribution of the study participants by hrHPV status and cytology diagnosis

	hrHPV detected n = 56						hrHPV not detected n = 115						
Age group	ASC-H	ASCUS	AEC	AEC-FN	LSIL	NILM	ASC-H	ASCUS	AEC	AEC-FN	LSIL	NLM	Total
20–30	0	1 (0.58)	0	0	0	9 (5.26)	0	0	1 (0.58)	0	2 (1.17)	19 (11.11)	32 (18.71)
31–40	0	0	0	0	2 (1.17)	9 (5.26)	1 (0.58)	2 (1.17)	0	1 (0.58)	1 (0.58)	31 (18.13)	47 (27.49)
41–50	0	3 (1.75)	0	0	1 (0.58)	16 (9.36)	2 (1.17)	1 (0.58)	1 (0.58)	0	2 (1.17)	22 (12.87)	48 (28.07)
50 <	1 (0.58)	0	0	0	0	14 (8.19)	0	1 (0.58)	0	0	0	28 (16.37)	44 (25.73)
Total	1 (0.58)	4 (2.34)	0	0	3 (1.75)	48 (28.07)	3 (1.75)	4 (2.34)	2 (1.17)	1 (0.58)	5 (2.92)	100 (58.48)	171 (100)

Fisher's exact test: Age group by hrHPV status, *P*-value = 0.304

Fisher's exact test: Age group by cytology diagnosis, *P*-value = 0.633

The *p*-value for the age groups by hrHPV status was 0.304, indicating no statistically significant association between age groups and hrHPV status. Similarly, the p-value for the age groups by final cytology diagnosis was 0.633, indicating no statistically significant association between age groups and final cytology diagnosis.

Table 5 presents an unadjusted logistic regression (adding risk factors individually to the model). Results shows that for every unit increase in age, women are equally likely to have hrHPV [95% CI = (0.96, 1.02), *p*-value = 0.754]. Women living with HIV were 0.85 less likely to have HPV [95% CI = (0.43, 1.70), *p*-value = 0.984]. Women who had more than 1 partner were 1.18 more likely to get hrHPV [95% CI = (0.49, 2.88), *p*-value = 0.708]. However, all *p*-values were reported to be more than 0.05, meaning these associations were statistically insignificant. After adjusting the logistic regression for multicollinearity by adding all risk factors in the model, the table shows that for every unit increase in age, women were still equally likely to have hrHPV [95% CI = (0.97, 1.03), *p*-value = 0.903]. Women living with HIV were 0.85 less likely to have hrHPV [95% CI = (0.35, 2.08), *p*-value = 0.647]. Women who had more than 1 partner were 1.28 more likely to get hrHPV [95% CI = (0.19, 8.83), *p*-value = 0.587]. All *p*-values were reported to be more than 0.05, meaning these associations were statistically insignificant.

**Table 5:** hrHPV risk association with age, HIV status and number of lifetime sexual partners

	**Risk factor**	**ODDS RATIO**	**95% CI**	**P-value**
	Age	1.00	0.96, 1.02	0.754
**Unadjusted**	HIV positive	0.85	0.43, 1.70	0.984
	More than 1 sexual partner	1.18	0.49, 2.88	0.708
	Age	1.00	0.97, 1.03	0.903
**Adjusted**	HIV positive	0.85	0.35, 2.08	0.647
	More than 1 sexual partner	1.28	0.19, 8.83	0.587

## Discussion

This study reports hrHPV prevalence of 32.7% (56/171) in HPV vaccine-naïve women who were 21 years and above from the indigenous and marginalized communities of Ghanzi and Kgalagadi regions in western Botswana. This is the first study to report hrHPV genotypes detected from cervical screening in a unique population of indigenous (marginalized) persons residing in the western region of Botswana. The median age of the participants was 42 years (Table 1); 74% (127/171) were aged 50 years and below while 26% (44/171) were above 50 years. Prevalence of pre-malignant cervical lesions in this study was 13.5% (Table 2) and the prevalence of hrHPV DNA in premalignant cervical lesions was 35%. Overall, the hrHPV prevalence was lower than that reported by a previous study in Botswana in unvaccinated young college women with a prevalence rate of 60.2% [33]. Other studies reported varying hrHPV prevalence rates between 30–64% mainly focused on women with premalignant and malignant cervical lesions and living with HIV [27, 34–36]. However, these studies focused on women living in and around Gaborone (see map above).

The hrHPV genotypes detected in premalignant cervical lesions included the quadrivalent vaccine targeted HPV18, (6.25%) and non-quadrivalent HPV 31, 35, 39, 51, 52 and 56 at 10.42%, 4.7%, 6.25%, 14.5%, 12.5% and 4.17% respectively (Table 3). HPV16 was only detected in women with normal cytology at a prevalence rate of 10.42%. This finding is in contrast with the observations that HPV16 is the most common genotype despite geographic variation for other HPV genotypes [37, 38]. High-risk HPV infection was also detected more in women with normal cervical cytology, followed by LSIL and ASCUS (Table 4). In addition, more hrHPV associated cervical premalignant lesions were detected in women less that 50 years (Table 4). There was an increased detection of non-quadrivalent targeted hrHPV genotypes in the ASCUS lesion as compared to other cervical premalignant lesions with HPV 35, 51 and 56 being the most commonly detected in this category. In 56 of the 171 women (32.7%) who tested positive for hrHPV DNA; seven women living without HIV had cervical premalignant lesions and 2/7 (14.2%) had hrHPV (HPV18, HPV39 and HPV56) with only one woman out of the two, having hrHPV genotypes that are targeted by the current quadrivalent vaccine that is in use in Botswana. Six out of seven (85.7%) women with cervical premalignant lesions had hrHPV genotypes which are non-quadrivalent vaccine targeted. Although the predominance of HPV genotypes 51, 52 and 56 in our study is not consistent with global findings, previous studies in Botswana have also reported a higher prevalence of HPV genotype 51; 15.9%[39], 15.9%[40] 12.6% [36] highlighting the importance of HPV51 genotype circulation in the general population. Similarly, quadrivalent-targeted hrHPV genotype prevalence in these studies ranged from 21% to 34.3% [36, 40]. It is also important to note that these studies have been conducted among women from urban and peri-urban areas (Gaborone and surrounding areas), who have access to better healthcare services. In our study quadrivalent vaccine targeted hrHPV genotype prevalence was 16.67% vs 83.33% of the non-quadrivalent vaccine targeted genotypes.

Risk factors including the number of lifetime partners, age, parity, smoking and contraceptive use were also assessed for association with hrHPV positivity. Our results indicate that smoking history was the only statistically significant risk factor for hrHPV detection in this cohort (Table 1) with a *p*-value of 0.01. Studies have reported smoking as a risk factor for HPV infection [41, 42] and this has been supported by our findings. Association between HIV and hrHPV positivity showed a marginal significance (p-value 0.06) in Table 1, with the data indicating that WLWH were 0.85 less likely to have HPV [95% CI = (0.43, 1.70), *p*-value = 0.984] as indicated in Table 5. However, all p-values were more than 0.05, meaning that the association between living with HIV and hrHPV positivity were statistically insignificant. Our study also reports a slightly higher HIV prevalence (31%), than the national prevalence estimated to be at 26.2% for adult women (15 – 49 years) living with HIV in Botswana, [23]. This prevalence however, is also higher than that reported by the Botswana AIDS Impact Survey for both Kgalagadi and Ghanzi regions, which ranged between 15.2 – 21.1% for women aged between 15–64 years [23]. In our study, hrHPV/ HIV co-infection was 37.2% (Table 1); cervical premalignant lesions were detected in 13.3% of WLWH in this study and 30.2% of the women with hrHPV had normal cytology. Another study by Luckett et al. [43], in a subset of WLHW and diagnosed with CIN2 + in Gaborone reported a hrHPV prevalence of 29% which was lower than that reported by our study. Women living with HIV have been reported to be more likely to have hrHPV infection than those living without HIV [44, 45]. Since HIV and hrHPV infections share a common route of transmission, it is to be expected that these findings corroborate those of previous studies of the duality of existence in their host [46]. HIV infection is a well-known risk factor for HPV persistence, cervical cancer development and its progression [47–50], with the burden of cervical cancer in Botswana reported to be largely driven by the high HIV prevalence [43]. This has been attributed to immune suppression which supports hrHPV persistence and ultimate cancer development [45, 48].

In this study, 23/171 (13.5%) of the women had cervical premalignant lesions. Of the 23, only 8 had hrHPV (8/23). In addition, 9/23 (39%) women with premalignant lesion were living with HIV. Nine women living with HIV had cervical premalignant lesions and 4/9 (44%) had hrHPV (HPV 31, HPV 52, HPV 56 and HPV 58) DNA. HPV 58 has been previously reported as the common type in WLWH with cervical premalignant lesions in Botswana [39]. The high hrHPV prevalence and low detection of cervical premalignant lesion in our study population could be explained by the success of highly active antiretroviral therapy (HAART) in Botswana. Although we did not assess the viral suppression, recent reports indicate that 98.4% of WLWH aged 15–64 years in Botswana are on antiretroviral treatment and 98.6% have viral suppression [23].

A total of 12/56 (21.4%) women in our study who were co-infected with more than one hrHPV genotype and 50% of them had HIV infection. In this study, the most common hrHPV genotypes detected were 52, 51, 31, and 16 respectively, in WLWH with only HPV 16 as the genotype being targeted by the current vaccine being administered in Botswana. A study by Kim et al., [51] reported that “*uncommon and rare HPV genotypes may provide incremental etiologic contributions in cervical carcinogenesis*.” The predominance of this pattern of hrHPV genotypes in this population corroborates findings from previous studies in Botswana, especially for people living with HIV and warrants further attention. The findings of this study highlight the geographic disparity of understudied and underrepresented populations in Botswana and add the dynamics of region-specific hrHPV distribution of varying carcinogenicity. The high HIV prevalence in this community also calls for consideration of healthcare policies to address cervical cancer surveillance, screening and treatment monitoring which may include region-specific HPV vaccine designs for prevention strategies in isolated populations. These hrHPV genotypes are not covered by the current prophylactic vaccine in use, which is achieving 90 -100% nationwide coverage and is expected to reduce cervical lesions caused by HPV16 and HPV18. Other studies in Botswana have also reported low hrHPV prevalence for quadrivalent targeted hrHPV genotypes [36, 40]. However, these studies were conducted on a younger cohort (18-22 years) for vaccine impact monitoring and lacked cervical cytology results for comparison.

## Limitations of the study

A smaller sample size of cervical premalignant lesions in our study could have introduced a bias. However, these are naturally smaller communities and isolated in nature. We also note that our findings may not be generalizable to other indigenous (marginalized) populations in Botswana accounting for the fact that there is cultural diversity among indigenous communities including those in Botswana. A major limitation of this study is that no high-grade lesions were detected, therefore there were no biopsies collected and analyzed. This study was conducted alongside routine Ministry of Health mobile screening outreach service guidelines, therefore all women with abnormal cytology results were referred for further management through their healthcare facility which also served as a study site. However, there are plans for a follow-up study in the future to assess treatment uptake in this very same community. To our knowledge, this is the first study to report hrHPV prevalence, diversity and cervical disease burden focused on a specific population of marginalized and indigenous populations of Botswana.

## Conclusions

Our study found a predominance of non-quadrivalent vaccine targeted hrHPV genotypes in the indigenous and marginalized communities in Botswana and this is atypical of what has been observed in other populations. Although our study has a small sample size of abnormal cervical cytology, we note that these hrHPV genotypes detected are epidemiologically significant. Marginalized communities’ geographical isolation and territorial relationships could provide a niche for these less common hrHPV genotypes to increase the burden of non-quadrivalent targeted vaccine hrHPV-associated cervical carcinogenesis. Therefore, there is a need to monitor and further investigate these findings and determine their potential impact on cervical carcinogenesis in this isolated population. Furthermore, these results support those of previous studies on the consideration of broadening protection against hrHPV genotypes that are not targeted by the current vaccine in use.

## Data Availability

All relevant data is available upon request from the corresponding author, Patricia S. Rantshabeng.
